# Calcium and Cadmium Activate ESRRB to Mediate Cell Stemness and Pluripotency

**DOI:** 10.3390/ijms27010231

**Published:** 2025-12-25

**Authors:** Xu Shi, Gai Yan, Nicole C. Zhao, Qiaochu Wang, Dajun Lu, Destiny Lawler, Reem M. Gahtani, Celia Byrne, Bassem R. Haddad, Robert L. Copeland, Mary Beth Martin

**Affiliations:** 1 Department of Biochemistry and Molecular and Cellular Biology, Georgetown University, Washington, DC 20007, USA; 2 Department of Oncology, Georgetown University, Washington, DC 20007, USA; 3 Department of Pharmacology, Howard University, Washington, DC 20059, USArlcopeland@howard.edu (R.L.C.); 4 Department of Preventive Medicine and Biostatistics, Uniformed Services University of the Health Sciences, Bethesda, MD 20814, USA; 5 Lombardi Comprehensive Cancer Center, Research Building, 3970 Reservoir Road NW, Washington, DC 20007, USA

**Keywords:** cadmium, calcium, ESRRB, stemness/pluripotency/differentiation, ligand binding domain

## Abstract

Estrogen-related receptor beta (ESRRB) is thought to be an orphan receptor that functions as a transcription factor, pioneer factor, and mitotic bookmarker to regulate cell stemness, pluripotency, and differentiation. This study (1) investigates whether calcium and cadmium activation of ESRRB regulates signaling pathways of stemness and pluripotency, (2) explores the transcriptomic and biological alterations of metal activation of ESRRB, and (3) reveals the underlying mechanisms by which metals activate ESRRB. In HEK293T cells, treatment with calcium and cadmium increased the expression of ESRRB-regulated genes that was blocked by an ESRRB antagonist. In the breast cancer cell line MDA-MB-453, treatment with calcium, cadmium, or a synthetic agonist also increased the expression of ESRRB-regulated genes that was blocked by the antagonist, enhanced ESRRB nuclear localization, increased the recruitment of RNA polymerase 2 to estrogen-related receptor response elements (ERRE), enhanced cell stemness and proliferation pathways, and induced the expression of estrogen receptor alpha (ESR1 or Erα). Mutational analysis and molecular docking identified potential metal interaction sites within ESRRB’s ligand-binding domain. Together, these results suggest calcium acts as a natural ligand for ESRRB and cadmium, which mimics calcium, activate ESRRB to mediate cell stemness and pluripotency.

## 1. Introduction

Stem cells exist in embryos and adult tissue and are capable of self-renewal and differentiation into different cell types. A core network of transcription factors maintains the stemness and pluripotency and include OCT4 (OCT3/4 or POU class 5 homeobox1), SOX2 (sex-determining region Y-box 2), KLF4 (Kruppel-like factor 4), and NANOG (Nanog homeobox) [1,2,3,4,5,6]. As part of the core network estrogen-related receptor beta (ESRRB, EsRRβ, or ERRB) functions as a pluripotency transcription factor, as a pioneer factor and a mitotic bookmarker. As a pluripotency transcription factor, ESRRB regulates the expression of the core transcription factors, including itself, and differentiation factors [2,6,7,8,9,10]. As a pioneer factor, ESRRB binds to silenced enhancers and establishes a permissive chromatin structure for the recruitment of the pluripotency factors OCT4, SOX2, and NANOG [7]. Mitotic bookmarking is a process in which some transcription factors remain bound to a subset of their targets during mitosis, enabling daughter cells to rapidly resume the transcription of key genes. During mitosis, ESRRB bookmarks enhancers and promoters of genes highly transcribed in stem cells including *SOX2* and *KLF4* [9,11].

Estrogen-related receptor beta belongs to the nuclear receptor subfamily 3 group B member 2 (NR3B2), the same subfamily as estrogen receptor alpha (ESR1, ERα, NR3A1) [12]. Similarly to other nuclear receptors, ESRRB consists of the A/B domain at the N-terminus, which contains the ligand-independent activation function-1 (AF-1); the C domain, which includes the DNA-binding domain (DBD); the D domain, which is the hinge region; the E domain, which contains the ligand-binding domain (LBD) and the ligand-dependent activation function-2 (AF-2); and the F domain at the C-terminal end [13,14]. There are three members of the ESRR family: ESRRA (ESRRα), ESRRB (ESRRβ), and ESRRG (ESRRγ). ESRRA and ESRRG are key regulators of metabolism [15,16,17,18,19] while ESRRB is a key regulator of self-renewal and pluripotency in stem and reprogrammed cells [2,7,8,10,20].

In contrast to many nuclear receptors, ESRRs are thought to be orphan receptors that are constitutively active in the absence of a ligand [10,18,21,22,23,24,25,26]. However, several studies report that cholesterol may be an endogenous agonist for ESRRA [27,28] and that an estradienolone-like steroid from the urine of pregnant women may be an antagonist of ESRRA and ESRRG which interacts with and inhibits the transcriptional activity of the receptors [29]. A coincidental finding also suggests that compounds or factors removed from charcoal-stripped serum modulate ESRRB activity [30] and another study reports that 4-methylenesterol from a marine sponge may be an antagonist of ESRRB [31]. We recently reported that calcium mediates the effects of glucagon on gluconeogenesis as a probable ligand of ESRRG [32]. Together, these observations raise questions about whether ESRRs are truly orphan receptors and provide novel insights into ESRR signaling.

Calcium plays a pivotal role in numerous physiological processes and cellular signaling pathways, including not only regulating cell pluripotency and differentiation, but also promoting cancer stem cells and therapy resistance [33,34,35,36,37,38,39]. Cadmium mimics calcium in biological systems due to its similar charge and ionic radius. We have shown that calcium is a physiological ligand of estrogen receptor-alpha, which mediates the cross-talk between epidermal growth factor and the ligand-binding domain of the receptor, and both calcium and cadmium activate ESR1 through an interaction with the ligand-binding domain of the receptor [40,41,42]. The structural and sequence homology between the ligand-binding domains of ESR1 and ESRRB suggests that calcium and cadmium may also be ligands of ESRRB. Additionally, we have shown in a rodent model that in utero exposure to environmentally relevant amounts of cadmium alters mammary gland development in female offspring. In utero exposure to the metal results in an expansion of the number of stem/progenitor cells in the neonatal mammary gland and subsequently increases the number of branches, basal epithelial cells, and density of the adult gland suggesting that cadmium disrupts mammary gland development [43,44]. Cadmium also induces the expression of cancer stem cell markers in the breast and liver cancer cell lineages and promotes the conversion of non-cancer stem cells into cancer stem cells [45]. These observations line up the evidence and bring up our interests on how calcium/cadmium regulates cell stemness.

The aim of this study is to investigate whether calcium and cadmium mediate cell stemness, pluripotency, and differentiation through the activation of ESRRB. Based on the role of ESRRB as a transcription factor, this study demonstrates this from the changes in ESRRB-regulated genes, the localization of ESRRB, the transcriptomic and biological alterations, to where and how metals interact with ESRRB to reveal the underlying mechanisms by which metals activate ESRRB. The results suggest that calcium is potentially a natural ligand of ESRRB and cadmium, which mimics calcium, activates ESRRB to regulate cell stemness, pluripotency, and differentiation. Identification of natural and environmental ligands of ESRRB may enhance our understanding of its function in regulating cell fate.

## 2. Results

### 2.1. Calcium and Cadmium Induce ESRRB-Regulated Genes in HEK293T Cells

ESRRB regulates the expression of the stem/pluripotent factors, SOX2, KLF4, NANOG, and OCT4, to maintain cell renewal and pluripotency. ESRRB also regulates the expression of SFRP2 (secreted frizzle protein 2), which is part of the Wnt signaling pathway that regulates cell growth and differentiation; PRL (prolactin), which is a secreted hormone that promotes lactation and regulates growth by suppressing apoptosis; IGF1 (insulin-like growth factor 1), which is involved in mediating growth and development; and DPT (dermatopontin, tyrosine-rich acidic matrix protein), which interacts with decorin to modify the behavior of tumor growth factor-beta to regulate cell proliferation [46].

**Figure 1:**
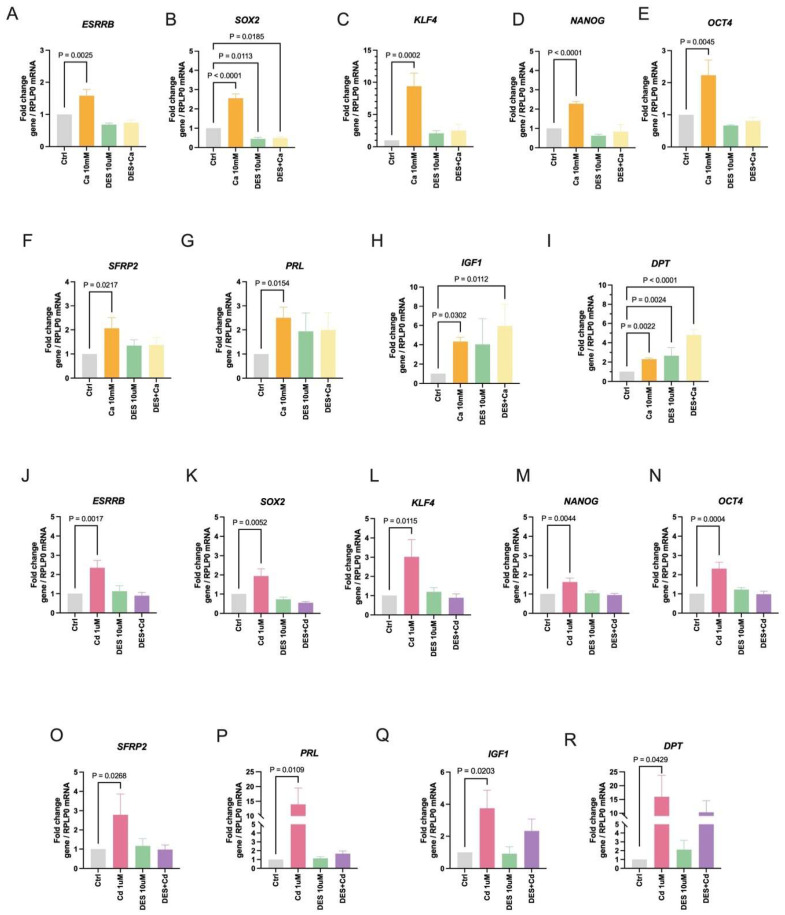
Effects of calcium and cadmium on ESRRB-regulated genes in HEK293T cells. (**A**–**E**), Effects of calcium on the induction of stemness core transcription factor genes *ESRRB*, *SOX2*, *KLF4*, *NANOG*, and *OCT4*. (**F**–**I**), Effects of calcium on the induction of *SFRP2*, *PRL*, *IGF1*, and *DPT*. (**J**–**N**), Effects of cadmium on the induction of stemness core transcription factor genes *ESRRB*, *SOX2*, *KLF4*, *NANOG*, and *OCT4*. (**O**–**R**), Effects of cadmium on the induction of *SFRP2*, *PRL*, *IGF1*, and *DPT*. Data are expressed as fold change (mean ± SEM); *n* = 3 to 7; statistical significance is defined as a *p* value of <0.05, *p* value is marked above if statistically significant.

To ask whether calcium and cadmium activate ESRRB, HEK293T cells were treated with calcium (10 mM) or cadmium (1 μM) in the absence or presence of the ESRRB inhibitor diethylstilbestrol (DES, 10 μM) and the mRNA of ESRRB-regulated genes was measured. Treatment with calcium for 24 h resulted in a significant increase in the expression of the stem/pluripotent factors *ESRRB* (~1.6-fold; Figure 1A), *SOX2* (~2.6-fold; Figure 1B), *KLF4* (~9.4-fold; Figure 1C), *NANOG* (~2.3-fold, Figure 1D), and *OCT4* (~2.3-fold, Figure 1E) and the differentiation genes *SFRP2* (~2.1-fold; Figure 1F), *PRL* (~2.5-fold; Figure 1G), *IGF1* (~4.4-fold; Figure 1H), and *DPT* (~2.3-fold; Figure 1I). DES, an ESRRB antagonist, blocked the calcium effects except for the *IGF1* and *DPT*. DPT upregulation by calcium in the presence of DES may be through the vitamin D receptor [47,48]. Similar to treatment with calcium, treatment with cadmium for 48 h resulted in a significant increase in the expression of *ESRRB* (~2.4-fold; Figure 1J), *SOX2* (~2.0-fold; Figure 1K), *KLF4* (~3.0-fold; Figure 1L), *NANOG* (~1.7-fold; Figure 1M), *OCT4* (~2.3-fold; Figure 1N), *SFRP2* (~2.8-fold; Figure 1O), *PRL* (~14.0-fold; Figure 1P), *IGF1* (~3.8-fold; Figure 1Q), and *DPT* (~16.0-fold; Figure 1R). DES blocked the effects of cadmium on *ESRRB*, *SOX2*, *KLF4*, *NANOG*, *OCT4*, *SFRP2*, *PRL*, *IGF1* and *DPT*. Taken together, the ability of calcium and cadmium, which mimics calcium, to increase ESRRB target gene expression and DES to block the increase suggests that calcium and cadmium activate *ESRRB*.

### 2.2. Calcium and Cadmium Induce ESRRB-Regulated Genes in MDA-MB-453 Cells

**Figure 2:**
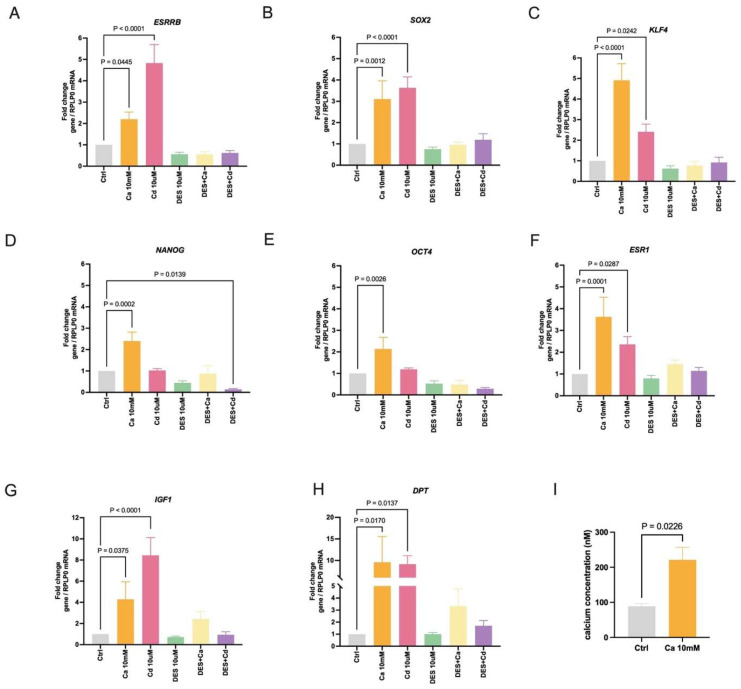
Effects of calcium and cadmium on ESRRB-regulated genes in MDA-MB-453 cells. (**A**–**E**), Effects of calcium and cadmium on the induction of stemness core transcription factor genes *ESRRB*, *SOX2*, *KLF4*, *NANOG*, and *OCT4*. (**F**–**H**), Effects of calcium and cadmium on the induction of *ESR1*, *IGF1*, and *DPT*; data are expressed as fold change (mean ± SEM); *n* = 5 to 7. (**I**), Effects of extracellular calcium treatment on the concentration of intracellular calcium. Data are expressed as calcium concentration in nM (mean ± SEM); *n* = 3. Statistical significance is defined as a *p* value of <0.05, *p* value is marked above if statistically significant.

To determine whether similar results are observed in a second cell line, MDA-MB-453 cells were treated with calcium (10 mM) or cadmium (10 μM) in the absence or presence of the ESRRB inhibitor DES (10 μM) for 24 h. MDA-MB-453 cells are a TNBC cell line that does not express *ESR1*, *SFRP2*, or *PRL*. Treatment with calcium resulted in a significant increase in the expression of stem/pluripotent factors *ESRRB* (~2.2-fold; Figure 2A), *SOX2* (~3.1-fold; Figure 2B), *KLF4* (~4.9-fold; Figure 2C), *NANOG* (~2.4-fold; Figure 2D), and *OCT4* (~2.2-fold; Figure 2E) and the differentiation genes *IGF1* (~4.3-fold; Figure 2G) and *DPT* (~9.6-fold; Figure 2H). However, MDA-MB-453 cells are classified as ERα negative breast cancer cells, and treatment with calcium induced the expression of *ESR1* (~3.6-fold; Figure 2F) that was blocked by DES. Treatment with the *ESRRB* agonist GSK4716 also induced the expression of *ESR1* (~2.4-fold; Supplemental Figure S1A), suggesting the role of *ESRRB* in the expression of *ESR1*. Although treatment with cadmium had no significant effect on the expression of *NANOG* (Figure 2D) and *OCT4* (Figure 2E), treatment with cadmium resulted in a significant increase in the expression of *ESRRB* (~4.9-fold; Figure 2A), *SOX2* (~3.4-fold; Figure 2B), *KLF4* (~2.4-fold; Figure 2C), *ESR1* (~2.4-fold; Figure 2F), *IGF1* (~8.5-fold; Figure 2G), and *DPT* (~9.2-fold; Figure 2H) and the increase was blocked by the *ESRRB* antagonist, similar to treatment with calcium. These results further support the role of calcium and cadmium in the activation of ESRRB. To activate the calcium-sensor receptor, calcium treatment (10mM) was used to increase intracellular calcium within physiological concentrations (Figure 2I). In MDA-MB-453 cells, 24 hr treatment with calcium increased the intracellular calcium concentration from approximately 88.8 (±13.0) nM to approximately 221.3 (±62.3) nM. This finding is consistent with our recent report, which showed that treatment of HEK293T cells with calcium increases the concentration of intracellular calcium from approximately 191 (±17) nM to 630 (±17) nM [32]. These results further support that calcium and cadmium increase gene expression through the activation of ESRRB.

### 2.3. Calcium and Cadmium Enhance the Localization of ESRRB

**Figure 3:**
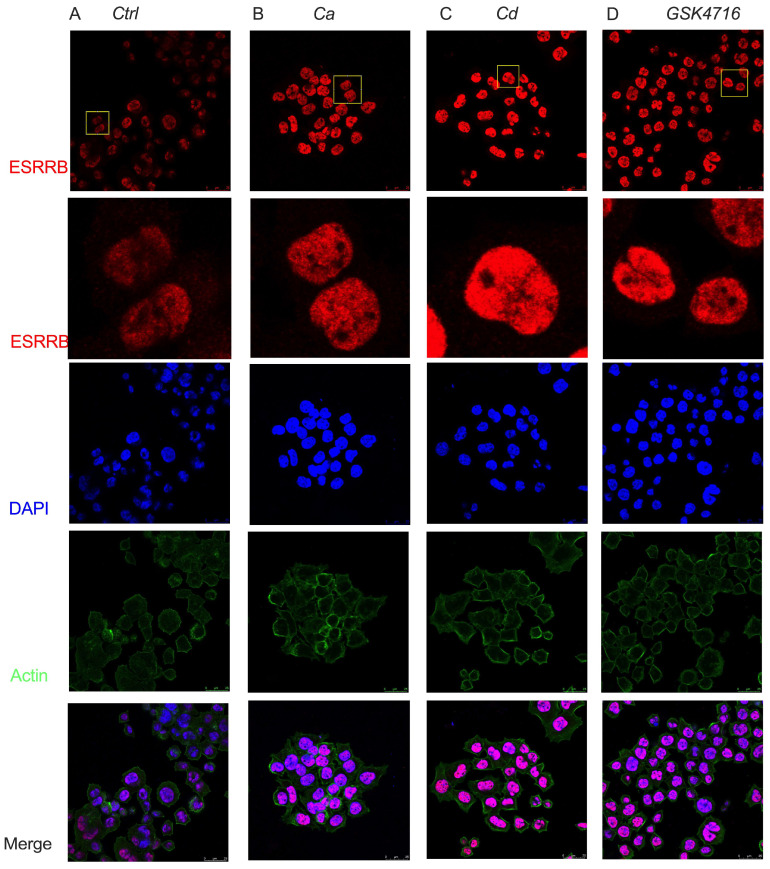
Effects of calcium and cadmium on ESRRB localization in MDA-MB-453 cells. (**A**–**D**), Immunofluorescence representative images of ESRRB in control (**A**), calcium (**B**), cadmium (**C**) and GSK4716 (**D**) treated cells. The second row of panels contains the zoomed-in images marked in yellow boxes in the top panels; red is ESRRB staining; blue is nuclear staining with DAPI; green is actin staining with phalloidin; bottom panels are merged IF pictures; the scale bar represents 25 μm.

The classical model of steroid hormone receptor activation has been extensively investigated, demonstrating that ligand binding to the receptor induces the dissociation of heat shock proteins, promotes receptor dimerization, and facilitates receptor translocation to the nucleus [49]. Although ESRRB belongs to the same subfamily as the steroid hormone receptor NSR1, the mechanism of activation of ESRRB may be different [12]. ESRRB functions as a canonical transcription factor that binds to an estrogen-related receptor response element (ERRE) and recruits RNA polymerase 2 (Pol2); a pioneer factor that binds to silenced DNA and recruits core pluripotency factors; and a mitotic bookmarker that remains bound during mitosis; [9]. As there is no known physiological ligand for ESRRB, the precise mechanisms governing ESRRB activation are not fully elucidated. To determine the effects of calcium and cadmium on the localization of ESRRB, MDA-MB-453 cells were treated with calcium (10 mM), cadmium (10 μM), or the ESRRB agonist, GSK4716 (5 μM). The localization of ESRRB was evaluated by immunofluorescence. In untreated control cells, ESRRB was localized in the nucleus and to a lesser extent, in the cytoplasm and at the nuclear periphery (Figure 3A). Following treatment with calcium, cadmium, or GSK4716 for 3 h, ESRRB was primarily localized in the nucleus (Figure 3B–D). The relocalization of ESRRB from the cytosol to the nucleus in treated cells is consistent with its function as a ligand-activated transcription factor, while the localization of ESRRB in the nucleus in untreated cells is consistent with its function as a bookmarking factor.

### 2.4. Effects of Calcium and Cadmium on the Binding of ESRRB to the Enhancers of ESRRB Target Genes

**Figure 4:**
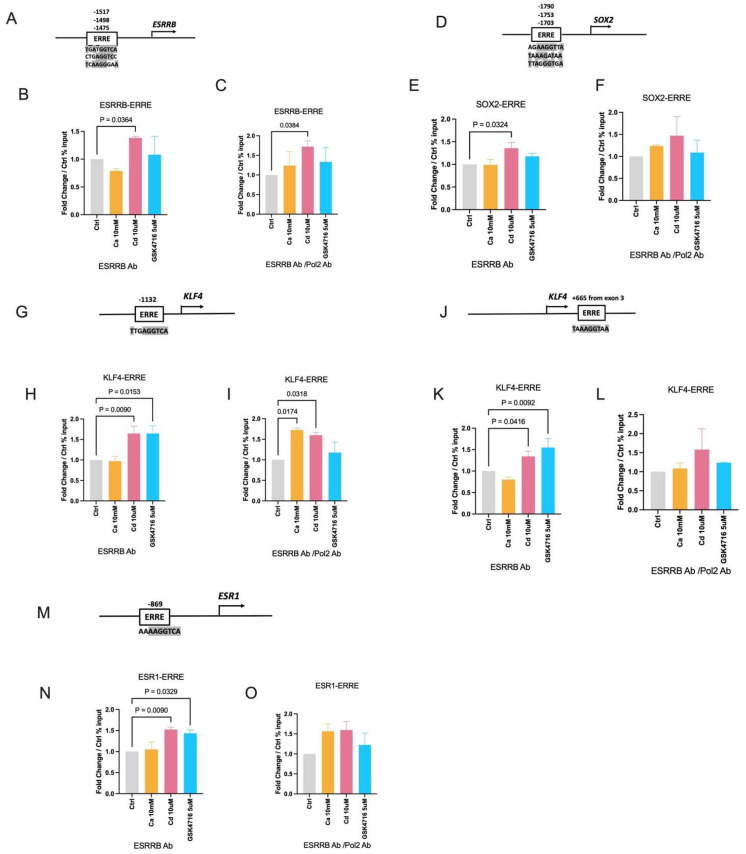
Effects of calcium and cadmium on the recruitment of ESRRB and RNA polymerase 2 to enhancers of ESRRB-regulated genes in MDA-MB-453 cells. (**A**–**O**), Effect of calcium, cadmium, and GSK4716 on the binding of ESRRB to the enhancer of *ESRRB* (**A**–**C**), *SOX2* (**D**–**F**), *KLF4* upstream enhancer (**G**–**I**) and intron enhancer (**J**–**L**), and *ESR1* (**M**–**O**). (**A**,**D**,**G**,**J**,**M**) illustrate the location of ERREs (estrogen-related receptor response elements) in the enhancer of target genes; the consensus sequences identical to classic ERRE (TNAAGGTCA) are highlighted in gray; the arrows indicate the transcription start site of each gene. (**B**,**E**,**H**,**K**,**N**) are the fold change in the binding of ESRRB to ERRE normalized to control (ChIP). Data are expressed as fold change in control in % input (mean ± SEM); *n* = 3. (**C**,**F**,**I**,**L**,**O**) are the fold change in the recruitment of Pol2 to ERRE to control (re-ChIP; first ESRRB pulldown then Pol2 pulldown). Data are expressed as fold change in control in % input (mean ± SEM); *n* = 2. Statistical significance is defined as a *p* value of <0.05, *p* value is marked above if statistically significant.

To characterize the transcriptional activity of ESRRB, MDA-MB-453 cells were treated with calcium (10 mM), cadmium (10 μM), or GSK4716 (5 μM) for 2 h and the binding of ESRRB to the enhancer regions of *ESRRB*, *SOX2*, *KLF4*, and *ESR1* was measured by a ChIP-qPCR assay and the recruitment of RNA polymerase 2 was measured by a re-ChIP-qPCR assay. By searching the UCSC Genome Browser, potential ERREs (TNAAGGTCA) were identified in the enhancer region of the target genes (Figure 4A,D,G,J,M). Calcium treatment did not result in a significant enrichment of ESRRB on the enhancer regions of the target genes. However, cadmium treatment resulted in a significant enrichment of ESRRB on the enhancers of *ESRRB* (~1.4-fold, Figure 4B), *SOX2* (~1.4-fold, Figure 4E), *KLF4* (upstream enhancer ~1.7-fold, Figure 4H and intron enhancer ~1.4-fold, Figure 4K), and *ESR1* (~1.6-fold, Figure 4N). Treatment with GSK4716 resulted in a significant enrichment on the enhancers of *KLF4* (upstream enhancer ~1.7-fold, Figure 4H and intron enhancer ~1.6-fold, Figure 4K) and *ESR1* (~1.5-fold, Figure 4N) but no enrichment on the enhancers of *ESRRB* (~1.1-fold, Figure 4B) or *SOX2* (~1.2-fold, Figure 4E). The ChIP-qPCR results with negative control IgG pull-down presented as yield are shown in Supplemental Figure S2.

To determine whether *ESRRB* recruited RNA polymerase 2 (Pol2) to the enhancers of target genes, a re-ChIP assay was performed. Although treatment with calcium did not increase the binding of ESRRB to the enhancers of *KLF4*, it increased the recruitment of Pol2 (~1.7-fold, Figure 4I) to the upstream enhancer of *KLF4*, which is located −1132 bp upstream of the transcription start site. Treatment with cadmium resulted in a significant increase in the recruitment of Pol2 to the enhancer regions of *ESRRB* (~1.8-fold, Figure 4C) and *KLF4* (upstream enhancer ~1.6-fold, Figure 4I). Treatment with cadmium also increased the recruitment of Pol2 to the enhancers of *SOX2* (~1.5-fold, Figure 4F), *KLF4* (intron enhancer ~1.6-fold, Figure 4L) and *ESR1* (~1.6-fold, Figure 4O) but the increases were not statistically significant. Although treatment with GSK4716 resulted in an increase in the binding of ESRRB to the enhancer regions of *KLF4* and *ESR1*, treatment with the agonist did not result in a significant increase in the recruitment of Pol2 to the enhancer regions of ESRRB target genes (Figure 4C,F,I,L,O). Taken together, the results are consistent with the functions of ESRRB not only as a canonical transcription factor but also as a pioneer and bookmarker transcription factor.

### 2.5. Calcium and Cadmium Mediate Cell Stemness/Pluripotency Through ESRRB

**Figure 5:**
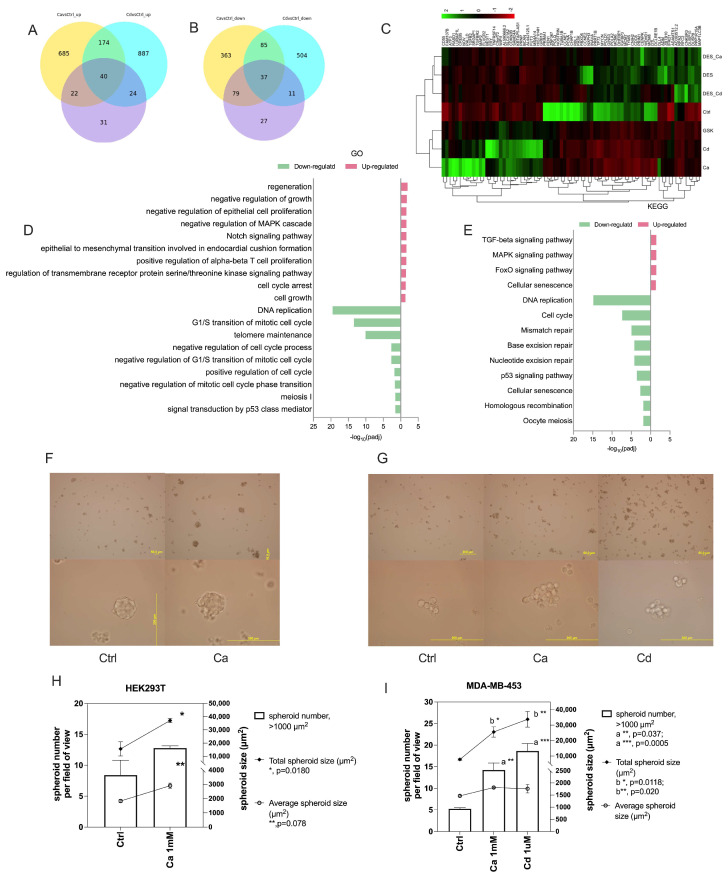
Effects of calcium and cadmium on cell stemness/pluripotency. (**A**), Venn diagram of up-regulated differentially expressed genes (DEGs) of Ca vs. Ctrl (in yellow balloon), Cd vs. Ctrl ( in blue balloon), and GSK vs. Ctrl groups (in purple balloon). (**B**), Venn diagram of down-regulated DEGs of Ca vs. Ctrl, Cd vs. Ctrl, and GSK vs. Ctrl groups. (**C**), Expression heat map for significant DEGs based on the FPKM value among groups; red, genes that were relatively up-regulated in groups; green, genes that were relatively down-regulated in groups; *n* = 3. (**D**,**E**), gene function enrichment analysis of the DEGs; red, gene functions were enriched in relatively up-regulated DEGs; green, gene functions were enriched in relatively down-regulated DEGs. Data are expressed as -log10 adjusted *p* value; *n* = 3. (**D**), Gene Ontology (GO) enrichment analysis of related biological processes. (**E**), Kyoto Encyclopedia of Genes and Genomes (KEGG) enrichment analysis of related pathways. (**F**–**I**), Effects of calcium or cadmium on spheroid formation in HEK293T and MDA-MB-453 cells. (**F**) (HEK293T cells) and (**G**) (MDA-MB-453 cells) are representative images of bright field in control, calcium, or cadmium treatment for 7 days. The scale bar represents either 50 μm or 200 μm as indicated. (**H**) (HEK293T cells) and (**I**) (MDA-MB-453 cells), quantification of calcium and cadmium treatment on spheroid-like formation. Only spheroids with an area ≥ 1000 μm^2^; were included in the analysis. Spheroid numbers were quantified in 6–8 randomly selected fields of view per experiment and expressed in bars refer to the left *y*-axis (mean ± SEM), as average and total spheroid size in dots with line refers to the right *y*-axis (mean ± SEM); Data were obtained from three independent experiments (*n* = 3); statistical significance is defined as a *p* value of <0.05; * *p* < 0.05; ** *p* < 0.01; *** *p* < 0.001.

To determine whether calcium and cadmium increase cell stemness, a spheroid formation assay was performed. HEK293T and MDA-MB-453 cells were plated in ultra-low attachment plates and treated with calcium (1 mM) or cadmium (1 μM) for 7 days. Treatment of HEK293T cells with calcium resulted in a significant increase in the average size and total size of the spheroids. The average size increased from 1832 ± 206 μm^2^ to 2907 ± 315 μm^2^ and the total size increased from 15,585 ± 9186 μm^2^ to 37,078 ± 2866 μm^2^ (Figure 5F,H). The total number of spheroids (spheroid size > 1000 μm^2^) increased from 8.4 ± 4.2 to 12.8 ± 0.6 but the increase was not significant (Figure 5H). Unlike HEK293T cells and other cancer cell lines, MDA-MB-453 cells form a relatively loose, spheroid-like structure (Figure 5G), as previously described [50,51]. Regardless of the morphological differences, treatment of MDA-MB-453 cells with calcium or cadmium resulted in a significant increase in the number of spheroids (spheroid size > 1000 μm^2^) from 5.3 ± 0.4 to 14.2 ± 2.8 and 18.7 ± 3.0, respectively, and the total spheroid size from 7791 ± 951 μm^2^ to 25,560 ± 5800 μm^2^ and 33,739 ± 8776 μm^2^, respectively. However, the average size of a spheroid only increased from 1467 ± 68 μm^2^ to 1809 ± 47 μm^2^ and 1763 ± 302 μm^2^, respectively (Figure 5G,I).

To investigate the cellular network of genes regulated by calcium and cadmium activation of ESRRB, RNA-seq was performed in MDA-MB-453 cells treated with calcium (10 mM), cadmium (10 μM), or the ESRRB agonist GSK4716 (5 μM) in the absence and presence of the ESRRB antagonist DES (10 μM) for 24 h. To investigate the function of overlapping regulated genes between the calcium, cadmium, and GSK4716 treatment, the differentially expressed genes (DEGs) of calcium versus control, cadmium versus control, and GSK4716 versus control were analyzed and compared. There were 77 DEGs in common between the calcium, cadmium, and GSK4716 treatment (listed in Supplementary Table S1); 40 DEGs were up-regulated (Figure 5A) while 37 DEGs were down-regulated (Figure 5B). The heatmap analysis of the transcriptional profile of the 77 DEGs (Figure 5C) shows that the calcium, cadmium, and GSK4716 groups have a similar pattern and are distant from the DES, calcium with DES, or cadmium with DES groups (also shown in Supplementary Figure S3A). In addition, Gene Ontology (GO) biological process terms such as regeneration, cell growth (including negative regulation of growth and cell growth), cell proliferation (including negative regulation of epithelial cell proliferation and positive regulation of alpha-beta T cell proliferation), negative regulation of MAPK cascade, Notch signaling pathways, regulation of transmembrane receptor protein serine/threonine kinase signaling pathway, and cell cycle arrest were enriched in the up-regulated DEGs (Figure 5D); while DNA replication, regulation of the cell cycle (including G1/S transition of mitotic cell cycle, negative regulation of the cell cycle process, negative regulation of the G1/S transition of the mitotic cell cycle, positive regulation of the cell cycle, and negative regulation of the mitotic cell cycle phase transition), telomere maintenance, meiosis 1, and signal transduction by p53 class mediator were enriched in the down-regulated DEGs (Figure 5D). The Kyoto Encyclopedia of Genes and Genomes (KEGG) pathway enrichment analysis using the list of up-regulated DEGs in calcium, cadmium, and GSK4716 treatment showed enriched signaling pathways, including TGF-beta, MAPK, and FoxO signaling pathways (Figure 5E). The KEGG analysis of the down-regulated DEGs showed an enrichment of pathways that included cell cycle, DNA repair (including mismatch repair, base excision repair, nucleotide excision repair, and homologous recombination), oocyte meiosis, and p53 signaling pathways (Figure 5E). Cellular senescence was enriched in both up-regulated and down-regulated DEGs, which reflects the complicated role of ESRRB in cell senescence. The results indicate that, similar to GSK4716, calcium and cadmium substantially alter the network of expressed genes through the activation of ESRRB in MDA-MB-453 cells. Taken together, the results suggest that calcium and cadmium activate the ESRRB-mediating biological processes and signaling pathways important for cell stemness/pluripotency.

### 2.6. Calcium and Cadmium Activate ESRRB Through Amino Acids in the Ligand-Binding Domain

**Figure 6:**
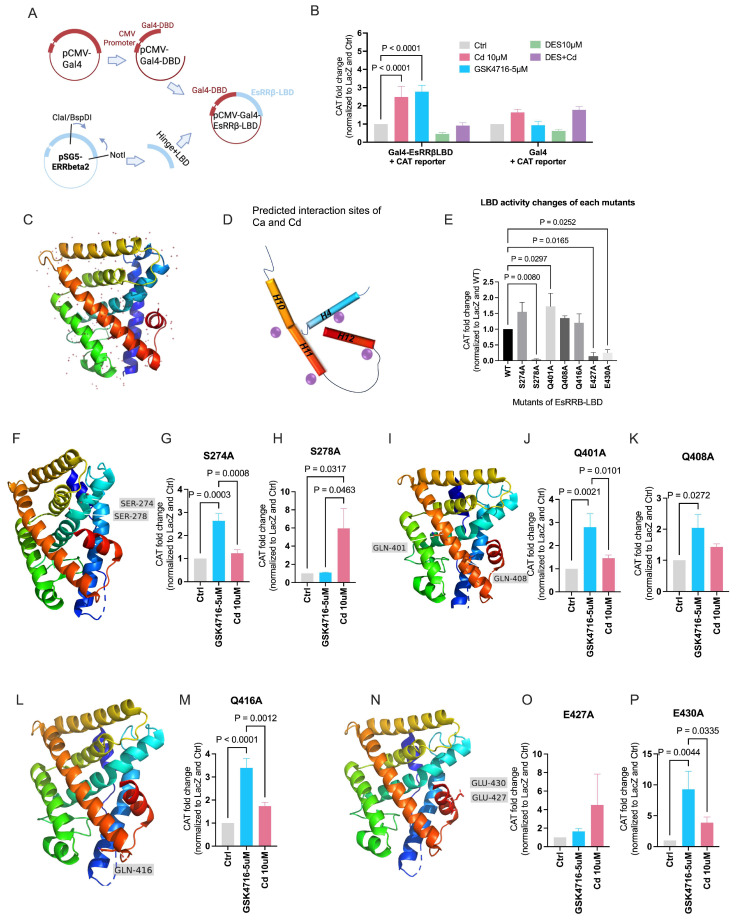
Identification of potential metal interaction sites on the LBD of ESRRB. (**A**), Chimeric GAL4-ESRRBsf-LBD construct. Created in BioRender. Shi, X. (2025) https://BioRender.com/a4hn9su (accessed on 16 October 2025). (**B**), Effect of cadmium and GSK4716 on WT LBD and GAL-4 plasmid. (**C**), Three-dimensional structure of ESRRB-LBD (6LN4); red dots represent H_2_O molecules. (**D**), Predicted interaction sites of calcium or cadmium on helix 4, 10,11 and 12; purple dots represent Ca^2+^ or Cd^2+^ at potential interaction sites. (**E**), CAT activity of GAL4-ESRRBsf-LBD mutants. (**F**,**I**,**L**,**N**) show the amino acid residues of predicted sites in 3D structure. (**G**,**H**,**J**,**K**,**M**,**O**,**P**), Effects of cadmium and GSK4716 on GAL-4-ESRRB-LBD mutants. Data are expressed as fold change (mean ± SEM); *n* ≥ 3; statistical significance is defined as a *p* value of <0.05, *p* value is marked above if statistically significant. LBD refers to ligand binding domain.

The LBD of ESRRB contains 11 alpha helices (H1, H3-H12) as do other nuclear receptors (Figure 6C). Our previous studies showed that calcium activates ESR1 through specific amino acids on the aqueous surface of the LBD of the receptor. The structural and sequence homology between the LBDs of ESR1 and ESRRB suggests that calcium and cadmium may activate ESRRB through a similar mechanism. There are three splice variants of ESRRB proteins in humans, which are the short-form (ESRRB-sf,433aa), beta 2 (ESRRB2, 500aa), and delta 10, (ESRRB-∆10, 508aa) [52,53]. ESRRB-sf is expressed in a broad range of human tissues and cells and has the strongest transcriptional activity, while ESRRB2 and ESRRB-∆10 are restricted to the testis and kidney with limited activity [54]. To ask whether metals activate ESRRB through the LBD, the chimeric plasmid, GAL4-ESRRBsf-LBD, was constructed by fusing the DNA-binding domain of GAL4 with the LBD of ESRRB-sf (Figure 6A). COS-1 cells, which do not express human ESRRB, were transfected with GAL4-ESRBBsf-LBD and a CAT reporter construct and treated with cadmium (10 μM) or GSK4716 (5 μM) in the absence or presence of DES (10 μM) for 24 h. Treatment with GSK4716 resulted in an approximately 2.8-fold significant increase in the expression of CAT. Treatment with cadmium also resulted in an approximately 2.5-fold significant increase in CAT that was blocked by DES (Figure 6B), suggesting that cadmium activates ESRRB through the LBD of the receptor.

Potential interaction sites in the LBD of ESRRB were identified by sequence alignment with the LBD of ESR1 (Figure 6D) [13], by mutational analysis, and by molecular docking. Sequence alignment identified S274, Q277, and S278 on helix H4; Q401, K405, and Q408 on helix H10 and H11; K414, Q416, and V419 on the C-terminal end of helix H11 and in the loop between H11 and H12; and H422/K423, E427, and E430 on helix H12. Based on the ability of cadmium and calcium to interact with amino acid side chains that contain sulfur, oxygen, or nitrogen [55], S274 and S278 on helix H4 (Figure 6F), Q401 and Q408 on helices H10 and H11 (Figure 6I), Q416 in the loop between helices H11 and H12 (Figure 6L), and E427 and E430 on helix H12 (Figure 6N) were identified and individually mutated to alanine in GAL4-ESRRBsf-LBD. The mutants were transiently transfected into COS-1 cells and treated with cadmium or GSK4716. The introduction of the S278A, E427A, and E430A mutations resulted in a significant decrease in the basal activity of the LBD of ESRRB. In contrast, the introduction of the Q401A mutation resulted in an approximately 1.8-fold increase in activity (Figure 6E). Similar to the wild type GAL4-ESRRBsf-LBD, treatment with GSK4716 resulted in an increase in the activity of GAL4-ESRRBsf-LBD S274A (~2.7-fold, Figure 6G), Q401A (~2.8-fold, Figure 6J), Q408A (~2.1-fold, Figure 6K), Q416A (~3.4-fold, Figure 6M), and E430A (~9.3-fold, Figure 6P) suggesting that mutation of S274, Q401, Q408, Q416, and E430 to alanine did not alter the ability of the LBD to activate transcription. However, treatment of the mutants with cadmium did not increase CAT expression suggesting that S274, Q401, Q408, Q416, and E430 are potential metal interaction sites in the LBD (Figure 6G,J,M,P). The S278A mutant, which had low basal activity, was not activated by GSK4716 but was activated by cadmium, indicating that S278 is important for the formation of the active conformation of the LBD by GSK4716 but not by cadmium (Figure 6H).

To verify the calcium/cadmium interaction sites on the ESRRB LBD, molecular docking was performed using AutoDock Vina (version 1.1.2). Given the unresolved inactive crystal structure of ESRRB without co-activators and the uncertainty regarding whether ESRRB functions as a monomer or a dimer, both the Apo-LBD (PDB ID: 6LN4) and Holo-LBD (PDB ID: 6LIT) crystal structures of ESRRB were used for docking simulations. The simulations were conducted with or without water molecules to account for the aqueous environment in which metal ion interactions typically occur. In agreement with the mutational analysis, molecular modeling using AutoDock Vina identified S274, Q401, and E430 as putative binding sites for calcium or cadmium on the ESRRB LBD, along with other potential interaction sites under varying conditions (Table 1).

Taken together, cadmium modulates ESRRB activity through its LBD. Amino acids S274 on helix H4, Q401and Q408 on helices H10 and H11, Q416 in the loop between helices H11 and H12, and E430 on helix H12 are potential interaction sites for cadmium and calcium.

The A chain or both chains of Apo and Holo structure models were used to identify the corresponding calcium or cadmium docking site via AutoDock Vina, with water considered as another factor in this molecular docking analysis.

## 3. Discussion

The role of ESRRB in stemness and pluripotency has been widely studied in embryonic stem cells and reprogrammed cells [2,7,8,10,20]. Increasing evidence suggests that calcium is involved in several key pathways that regulate cell renewal and pluripotency, such as Wnt, Hedgehog, Notch, TGF-beta, and FGF signaling pathways [33,38,56,57]. This study broadens our understanding of how calcium contributes to the regulation of cell self-renewal and pluripotency by activating ESRRB. The results show that calcium is a natural ligand of ESRRB and cadmium, which mimics calcium, and activates ESRRB to mediate cell fate. Calcium and cadmium increase the expression of ESRRB target genes in HEK293T and MDA-MB-453 cells. Further, the metals enhance the localization of ESRRB to the nucleus, the binding of ESRRB to estrogen-related receptor response elements, and the recruitment of RNA polymerase 2, thereby inducing target genes such as *ESRRB* and *KLF4* that are critical for cell pluripotency. Additionally, pathways that are related to cell pluripotency and proliferation, such as Notch, TGF-beta, and MAPK signaling pathways, are enriched following treatment with calcium, cadmium, or an ESRRB agonist. Moreover, S274, Q401, Q408, Q416, and E430 in the ligand-binding domain of ESRRB are potential interaction sites for calcium and cadmium, providing an insight into the underlying mechanisms by which metals active ESRRB.

Similarly to other members of the ESRR family of nuclear receptors, no endogenous ligands have been identified for ESRRB suggesting that ESRRB is an orphan nuclear receptor with constitutive activity [10,18,21,22,23,24,25,26]. However, several studies suggest that ESRRB is not constitutively active. To date, the apo structure of ESRRB has not been solved in the absence of a coactivator [16,25,58] and antagonists of ESRRB, such as diethylstilbestrol, inhibit the basal activity of the receptor. In addition, factors or compounds present in serum modulate ESRRB activity as evidenced by changes in ESRRB-regulated mRNA profiles [30]. We show that calcium activates ESRRB and that mutation of the putative metal interaction sites in the LBD decreases basal activity without altering the ability of a synthetic agonist to activate the receptor. Based on these observations, we argue that calcium is a ligand of ESRRB and a second messenger that mediates the effects of serum on the activity of ESRRB.

Calcium plays a vital role in numerous physiological processes including cellular signaling pathways. To maintain calcium homeostasis, cells regulate intracellular calcium concentrations within narrow limits [34,35,39]. In normal cells, the resting concentration of intracellular calcium is approximately 100 nM. Following activation, the concentration of intracellular calcium increases approximately 10- to 100-fold. Similarly to the control of intracellular calcium, the concentration of extracellular calcium is controlled to prevent systemic toxicity. In serum, the physiological concentration of calcium ranges from 1.0 to 2.5 mM. In hypercalcemia, the concentration is greater than 2.5 mM. To guard against hypercalcemia, cells regulate calcium uptake through calcium-activated channels. Cells in culture also take up calcium from the extracellular environment when the concentration of calcium reaches the threshold to activate the calcium-sensing receptor or when the ligand-gated and voltage-gated calcium channels are activated. To trigger calcium uptake in this study, the estrogen receptor-negative breast cancer cells were treated with millimolar concentrations of calcium resulting in an approximately 2.5-fold increase in intracellular calcium from ~89 nM to ~221 nM. Similar results were observed in estrogen receptor-positive cells treated with epidermal growth factor or high concentrations of extracellular calcium [40]. Cadmium is a heavy metal that has no physiological function and accumulates in the body due, in part, to its long biological half-life and the lack of efflux transporters [59]. Cadmium mimics calcium due to its similar charge and ionic radius but has a higher affinity than calcium for binding to some calcium targets [60]. For example, in estrogen receptor positive breast cancer cells, calcium binds to estrogen receptor alpha with a Kd of 500 × 10^−9^ M [40], whereas cadmium binds to estrogen receptor alpha with a Kd of 0.5 × 10^−9^ M [61]. The higher binding affinity of cadmium disrupts signaling pathways and the normal function of calcium targets [62].

The ligand-binding domain (LBD) of ESRRB exhibits structural homology with other nuclear receptors [63,64,65,66,67,68,69], folding into an antiparallel α-helical sandwich composed of 11 α-helices (H1, H3-H12) (Figure 6C). The ligand-binding pocket forms the LBD’s central core, enclosed by helices H5/6, H9, and H10. These helices are situated between two layers formed by H1–4, H7, H8, and H11 with helix H12 positioned adjacent to the ligand-binding pocket [68]. The activation mechanism is commonly described using Retinoid X receptor-alpha (RXRα) as the s paradigm, since its structure has been solved in the absence and presence of its ligand [63,70]. In the absence of a ligand, helices H10 and H11 are separated by a short loop. Helix H11 oriented almost perpendicular to helix H10 and pointing towards the ligand-binding pocket, while Helix H12 (contains the AF-2 activation domain), extends away from the LBD. In the presence of a ligand, helix H11 rotates to merge with H10 into a continuous helix, and H12 is strategically repositioned to cover the ligand-binding pocket. The reorganization leads to the formation the dimerization domain (helices H8 and H9, and the new helix H10/11) and the AF-2 domain (helix H3, H4, the loop H3 and H4 loop, and helix H12).

In proteins, metals have different functions including structural stabilization and conformational modulation. Through interactions with different amino acids, metals promote the local folding or assembly of different regions of the proteins into one domain [55]. Calcium has been reported to interact with the -OH group and -NH group of organic compounds [71] and has a preference for interacting with the side chains of asparagine, aspartic acid, glutamine, and glutamic acid, as well as main-chain carbonyl O atom, and sometimes interacts with the side chains of serine and threonine [72]. Similar to calcium, cadmium is a metal that is capable of forming tetrahedral or octahedral coordination complexes with the side chains of amino acid that contain sulfur, oxygen, or nitrogen, e.g., cysteine, serine, glutamic acid, aspartic acid, tyrosine, methionine, lysine, glutamine, asparagine, histidine, and threonine. Cadmium has also been shown to interact with the peptide oxygen of several residues, e.g., phenylalanine, lysine, arginine, asparagine, histidine, and glutamine [55].

Considering the structural homology of the ligand-binding domains of nuclear receptors, the established metal–amino acid interactions, and the results of the mutational analysis and molecular modeling, four potential metal binding sites are proposed in the ESRRB LBD. These proposed sites involve Q401, K405, and Q408 at the interface of helices H10 and H11; K414, Q416, and V419 on the C-terminal end of helix H11 and in the loop between helices H11 and H12; K423, E427, and E430 on the N-terminal end of helix H12; and S274, Q277, and S278 on helix H4. The interaction of calcium and cadmium with amino acids on helices that are proposed to undergo a conformational change when nuclear receptors are activated suggests a model whereby metals facilitate similar conformational changes in the ligand-binding domain of ESRRB [40]. Specifically, the model proposes that the interaction of calcium or cadmium with Q401, K405, and Q408 located at the C-terminal end of helix H10, between helices H10 and H11, and at the N-terminal end of helix H11 promotes the rotation of helix H11 around its axis and the formations of a continuous helix with helix H10 to form the dimerization domain. A similar helical rearrangement is observed in *pseudomonas* in the activation of I.3 lipase [73]. In the inactive conformation, a lid covers the active site of the enzyme. The lid is formed by a bent helix-turn-helix-motif that is similar to the structure of helices H10 and H11 in the apo-RXR-α. Calcium binding to an aspartic acid on the N-terminal helix of the first helix and to an aspartic acid on the C-terminal end of the second helix stabilizes a continuous helical structure between the two helices effectively opening the lid to the active site of lipase. The model of ESRRB activation also proposes that the two sites formed by K414, Q416, and V419 on the C-terminal end of helix H11 and in the loop between helices H11 and H12 and by K423, E427, and E430 on the N-terminal end of helix H12, facilitate the closure of helix H12 over the ligand-binding pocket. The interaction of metals at the two sites would alter the stability of helices H11 and H12 and induce a conformational change in the loop between H11–H12 closing helix H12 over the binding pocket to create the AF-2 domain. The model further proposes that the site formed by S274, Q277, and S278 on the helix H4 facilitates the coactivator recruitment to the AF-2 domain [66]. Although it is expected that most of the interactions between the metals and amino acids are with the side chains, it is also plausible that calcium and cadmium interact with the peptide oxygen, e.g., with valine. However, in support of this model, the amino acids S274, S278, Q401, and E430 are conserved in the ESRR family. E427 is conserved between the ESRR family and ESR1. Nevertheless, obtaining the crystal apo-structure of ESRRB in the absence of cofactors or ligands is crucial for accurately elucidating the mechanism of its activation. Further studies are needed to definitively validate this model.

The mammary gland is an organ that undergoes branching morphogenesis during puberty, lobuloalveolar expansion during pregnancy, and involution following lactation. The complex and cyclical nature of mammary gland development is predominantly conferred by mammary stem cells (MaSCs) [74] that give rise to a common progenitor cell, which differentiates into either basal/myoepithelial or luminal progenitor cells, which in turn commit to mature basal, luminal, and alveolar cells [75,76,77]. It has been suggested that breast cancer is due to perturbations in the normal development of the mammary gland giving rise to distinct subtypes of breast cancer. The basal/myoepithelial progenitor cells are thought to be the precursors of metaplastic/claudin-low breast cancers whereas the luminal progenitor cells are thought to be the precursor cells of basal-like, HER2 positive, and ER positive luminal A and luminal B breast cancers. The fate of the luminal progenitor cells is controlled by signaling pathways including Cripto-1, which is upstream of MAPK, Notch/CSL, and Wnt/β-catenin pathways [76]. Our GO and KEGG pathway enrichment analysis of up-regulated DEGs shows a significant enrichment of Notch (regulating proliferation, cell fate, differentiation, and cell death), TGF-beta (regulating cell proliferation, differentiation, apoptosis, cell plasticity), MAPK (modulating cell proliferation), and FoxO (a tumor suppressor pathway) pathways. Enrichment of the MAPK pathway following ESRRB activation by calcium, cadmium, or the ESRRB agonist is consistent with the enrichment of the MAPK pathway when ESRRB is overexpressed [78]. These findings support that metals have the same function as an ESRRB agonist that regulates cell stemness/pluripotency. The enrichment of the FoxO pathway in up-regulated DEGs is also consistent with the role of ESRRB as a tumor suppressor in breast and prostate cancers, as well as the association of ESRRB overexpression with improved prognosis [79,80,81,82]. Several mechanisms have been proposed for ESRRB’s tumor-suppressive effects, including the induction of cancer cell apoptosis, arrest of cell proliferation in the G1 phase, and inhibition of epithelial–mesenchymal transition (EMT). Interestingly, ESRRB has been shown to promote cell proliferation by suppressing the TGF-beta signaling pathway in cervical cancer [83]. Our results show the enrichment of the p53 signaling pathway in down-regulated DEGs following treatment with metals or the ESRRB agonist. The p53 is not only a tumor suppressor, but plays a critical role in switching off pluripotency during differentiation and is crucial in both pluripotent stem cells and pluripotent cancer cells [84]. These findings provide an integrative view of ESRRB, demonstrating its diverse roles across different cancer types.

Further, ESRRB functions not only as a transcription factor [8], but also as a pioneer factor, establishing permissive chromatin structures for other pluripotency factors [7], and a bookmarker transcription factor, remaining bound to enhancers and promoters of target genes during mitosis in stem cells [9]. Our observations in a breast cancer cell line support the multifaceted roles of ESRRB, where ESRRB demonstrated a similar pattern of binding and activity. Immunofluorescence staining validates the predominant nuclear localization of ESRRB, with augmented localization following treatment with metals or the agonist GSK4716. ChIP and re-ChIP results suggest that cadmium not only increased ESRRB binding to the enhancer regions of *ESRRB* and *KLF4* (−1132 bp from TSS), but also activated ESRRB transcriptional activity by recruiting Pol2, which is consistent with the role of ESRRB as a transcription factor. On the other hand, calcium treatment did not augment ESRRB binding to the enhancer region of *KLF4* (−1132 bp from TSS). Still, it increased the transcriptional activity of ESRRB, which is consistent with the role of ESRRB as a bookmarker transcription factor of the *KLF4* gene. Treatment with cadmium and GSK4716 increased ESRRB binding to the enhancer regions of *SOX2*, *KLF4* (+665 from exon 3), and *ESR*1, but did not increase the recruitment of Pol2, supporting the role of ESRRB as a pioneer transcription factor.

There is increasing evidence of discordance in estrogen receptor (ER, ESR), progesterone receptor (PGR) and human epidermal growth factor 2 (HER2/neu) status between primary breast tumors and metastatic and recurrent breast cancers, potentially impacting treatment options and ultimately affecting survival [85,86,87]. Discordance occurs most frequently in luminal A breast cancer followed by triple negative, luminal B, and HER2 cancers. The rate of discordance of ER expression between the primary tumor and the metastasis is low with loss of ER expression greater than the gain of ER expression [86]. Although low, there is evidence that triple-negative tumors may gain expression of ER during metastasis. MDA-MB-453 cells were originally isolated from a patient with TNBC but then categorized as HER-2 positive breast cancer cells—still estrogen receptor alpha-negative. Our findings demonstrate that cadmium enhances the recruitment of ESRRB to the ERRE sites in the ESR1 gene and regulates ESR1 expression, suggesting that ESRRB may play a role in the fate of luminal progenitor cells in normal development and tumorigenesis. Although further investigation is needed to elucidate the role of ESRRB in TNBC, ESRRB may be a promising therapeutic target in breast cancer.

This study investigates ESRRB activation by calcium and cadmium using HEK293T and MDA-MB-453 cells, explores the transcriptomic alterations of metal activation of ESRRB, finds an induction of estrogen receptor alpha, and identifies the potential interaction sites of calcium and cadmium with the ligand-binding domain of ESRRB. Although ESRRB is a multifaceted transcription factor with diverse roles in several cellular processes, its role in cancer remains ambiguous with research predominantly conducted in rodent embryonic and reprogrammed cells due to its function as a stemness/pluripotency factor. Studies involving cancer patients have been limited to specific cancer types, revealing differential roles and outcomes. A comprehensive understanding of the relation between stemness, pluripotency, differentiation, and tumorigenesis requires studies in a wider range of cancers than currently available. Moreover, further investigations using normal human cells, particularly adult stem cells, are essential to elucidate the role of ESRRB in tumor initiation.

## 4. Materials and Methods

### 4.1. Cell Culture

HEK293T (RRID: CVCL_0063) cells were acquired from American Type Tissue Culture. MDA-MB-453 (RRID: CVCL_0418) cells and COS-1 (RRID: CVCL_0223) cells were acquired from the Tissue Culture and Biobanking Shared Resource (Georgetown University, Washington, DC, USA). For cell growth and passaging, HEK293T cells were maintained in phenol red-free Improved Minimal Essential Medium (IMEM; Corning, Corning, NY, USA) containing 10% fetal bovine serum (FBS; Sigma-Aldrich, St. Louis, MO, USA). MDA-MB-453 cells were maintained in Dulbecco’s Modified Eagle’s Medium (DMEM; Corning, Corning, NY, USA) containing 10% FBS. For assays, 5 × 10^5^ HEK293T cells or 3 × 10^5^ COS-1 cells were plated in six-well plates with 3 mL of lipoic-acid and phenol-red free IMEM (Crystalgen, Commack, NY, USA or VitaScientific, College Park, MD, USA) containing 5% charcoal-stripped calf serum (CCS; Sigma-Aldrich, St. Louis, MO, USA) for 24 h; 6 × 10^5^ MDA-MB-453 cells were plated in six-well plates with 3 mL of DMEM containing 10% FBS overnight, the media was changed to 3 mL of lipoic-acid and phenol-red free IMEM (Crystalgen, Commack, NY, USA) containing 5% CCS for 24 h. For any treatment, the serum was then diluted to 1% CCS in 5 ml IMEM or the media was replaced by 5 ml IMEM (Crystalgen, Commack, NY, USA) without serum and cells were treated with 10 mM calcium, 1 or 10 µM cadmium, or 5 µM GSK4716 (in ethanol) in the absence or presence of 10 µM DES (in ethanol) for 2 h, 24 h or 48 h.

### 4.2. Real-Time-qPCR

Following treatment, RNA was isolated using Trizol (Life Technologies, Carlsbad, CA, USA), chloroform (Fisher Scientific, Waltham, MA, USA) and isopropanol (Fisher Scientific, Waltham, MA, USA). The reverse transcription reaction and qPCR assays were performed as previously described [32,88]. qPCR results were normalized to the ribosomal protein P0 (RPLP0) mRNA (Taqman assay) or β-galactosidase mRNA (SyBr Green assay) and presented as fold change compared to control by the 2^−ΔΔct^ method. The probes of the Taqman assay are listed in Supplemental Table S2.

### 4.3. Intracellular Calcium Measurement

The intracellular calcium was measured using fluorescence assay as previously described [32]. Briefly, MDA-MB-453 cells were treated with 10 mM calcium for 2 h or 24 h in IMEM. After treatment, the cells were trypsinized and washed with Hank’s balanced salt solution (HBSS; Gibco, Billings, MT, USA). They were then incubated in Fluo-4 AM (Invitrogen) dye solution at 37 °C for 30 min. A sample of 5 × 10^5^ cells were washed with HBSS and used for fluorescence measurements on a fluorimeter (Photon Technology International, Lawrenceville, NJ, USA), with an excitation wavelength of 494 nm and an emission wavelength of 516 nm.

### 4.4. Immunofluorescence

Immunofluorescence was performed as previously described [32,89], but using anti-ESRRB antibody for staining. Briefly, MDA-MB-453 cells were plated on 18 mm circular coverslips in 12-well plates (2 × 10^5^ cells per well) in DMEM containing 10% FBS for overnight. After cell adherence, the media were changed to lipoic-acid and phenol-red free IMEM containing 5% CCS for 24 h. Following the treatment with 10 mM calcium, 10 μM cadmium or 5 μM GSK4716 for 2 h, cells were washed with PBS and fixed with 4% formaldehyde (Fisher Scientific, Waltham, MA, USA) for 20 min and permeabilized using 0.5% Triton X-100 in PBS for 5 min. The fixed cells were blocked with 3% bovine serum albumin (BSA; Fisher BioReagents, Waltham, MA, USA) in PBS for 1 h. Cells were incubated with 1 µg/mL anti-ESRRB antibodies (PP-H6705-00, R&D Systems, Minneapolis, MN, USA; 1:1000 in 3% BSA) at 4 °C overnight and then with goat anti-mouse IgG polyclonal antibody (594; Alexa Fluor, Invitrogen, Waltham, MA, USA; RRID: AB_2313921, 1:500) at room temperature for 1 h. F-actin was stained with Phalloidin-iFluor Conjugate (488; AAT Bioquest, Pleasanton, CA, USA; 1:500) at the same time as IgG for 1 h at room temperature. The nuclei were stained with 0.5 μg/mL 4′,6′-diamidine-2-phenylindole dihydrochloride (DAPI; Sigma-Aldrich, St. Louis, MO, USA) for 5 min. The images were captured by Leica SP8 laser confocal microscope (Leica, Wetzlar, Germany).

### 4.5. Chromatin Immunoprecipitation (ChIP) and Re-ChIP

ChIP assay was performed with 1 × 10^7^ MDA-MB-453 cells per ChIP. After the treatment with 10 mM calcium, 10 µM cadmium or 5 µM GSK4716 in lipoic-acid and phenol-red free IMEM with 1% CCS for 2 h, cells were crosslinked with 1% formaldehyde and sonicated until the chromatin size was reduced to 200–300 bp. The remainder of the ChIP protocol was performed following the protocol described previously [90], using buffers purchased from Sigma Aldrich (20-153 Chromatin Immunoprecipitation Dilution Buffer; 20-154 Low Salt Immune Complex Wash Buffer; 20-155 High Salt Immune Complex Wash Buffer; 20-156 LiCl Immune Complex Wash Buffer). The antibodies were used at a final concentration of 5 μg antibody per 30 μg of chromatin and included ESRRB (PP-H6705-00, R&D Systems, Minneapolis, MN, USA), Pol2 (MA1-26249, Thermo Fisher Scientific, Waltham, MA, USA; RRID: AB_79535), and control mouse IgG (ab18413, Abcam, Cambridge, UK; RRID: AB_2631983) for the respective pulldowns. Precipitated DNA was recovered using the PCR purification kit (QIAquick PCR purification kit, QIAGEN, Germantown, MD, USA) and amplified by RT-qPCR using SYBR green mastermix (PowerUP SYBR Green Master Mix, Thermo Fishier Scientific, Waltham, MA, USA). The re-ChIP was performed using the protocol as previously described [91]. The protein-DNA complexes from the ChIP assay were isolated using the reChIP Elution buffer. The complexes were diluted using ChIP dilution buffer and followed by pulldown with antibodies against Pol2 antibodies, respectively. Mouse IgG was used as a negative control.

To find ERRE sites in target genes, UCSC Genome Browser was used to identify potential ERRE sites in the promoter and enhancer regions of *ESRRB*, *SOX2*, *KLF4*, and *ESR1*. TNAAGGTCA was used as the template of the ERRE sequence. The location of potential ERREs was also verified by the alignment of the human and mouse genomes based on the ChIP-seq data of the mouse on Cistrome Data Browser (http://cistrome.org/db/#/ (accessed on 20 August 2021), CistromeDB: 76697, 73564, 93,946 and 408) [9,92,93,94]. The ChIP primer sequences are listed in Additional Table S2.

### 4.6. Spheroid Formation Assay

HEK293T cells or MDA-MB-453 cells (0.5 × 10^5^ cells per well) were seeded into low-attachment 6-well plates and cultured in 3 mL of lipoic-acid- and phenol-red-free IMEM (Crystalgen, Commack, NY, USA). Cells were treated with either 1 mM Ca^2+^ or 1μM Cd^2+^ and maintained under these conditions for 7 days. On day 7, spheroid formation was documented using an Olympus IX71 inverted microscope (Olympus, Tokyo, Japan ). Quantitative analysis of spheroid formation was performed using ImageJ (version 1.53p). Only spheroids with an area ≥1000 μm^2^ were included in the analysis. Spheroid numbers were quantified in 6–8 randomly selected fields of view per experiment. Data were obtained from three independent experiments (*n* = 3).

### 4.7. Chloramphenicol Acetyltransferase (CAT) Reporter Assay

The plasmid for ESRRB variant 2 (pSG5-ERRbeta2) was a gift from Dr. Rebecca Riggins, Georgetown University (Addgene plasmid #52186; RRID: Addgene 52186; Addgene, Watertown, MA, USA) [53]. The plasmid containing GAL4 (pCMV-GAL4) was a gift from Liqun Luo (Addgene plasmid #24345; RRID:Addgene_24345; Addgene, Watertown, MA, USA) [95]. The β-galactosidease (pEQ176) plasmid was a gift from Adam Geballe (Addgene plasmid #83943; RRID:Addgene_83943; Addgene, Watertown, MA, USA) [96] and the GAL4-CAT reporter plasmid pG6(5′Pro) (P#1477) containing the chloroamphenicol acetyltransferase gene was a gift from Matija Peterlin (Addgene plasmid #14659; RRID:Addgene_14659; Addgene, Watertown, MA, USA) [97].

To create the GAL4-ESRRBsf-LBD plasmid, the hinge region and ligand-binding domain (LBD) of ESRRB (L169-A433) were copied by PCR from pSG5-ERRbeta2. A stop codon (taa) was added to the 3′ terminus by PCR assay and amino acid A433 was mutated to valine (gtg) to mimic the LBD of ESRRB short form (ESRRBsf) using the QuikChange Primer Design of Agilent. The restriction enzyme cut site for ClaI/BspDI was added to the 5′ terminus and NotI was added to the 3′ terminus. pCMV-GAL4 was cut with BspDI and NotI (New England Biolabs, Ipswich, MA, USA) and fused with the LBD of ESRRBsf. The GAL4-ESRRBsf -LBD plasmid was verified by sequencing.

### 4.8. Mutagenesis and Transfection Assays

To create GAL4-ESRRBsf-LBD mutants, the primers were designed using the QuikChange Primer Design of Agilent and synthesized by Integrated DNA Technologies. Plasmids and probes used are listed in Table 2. The primer sequences are listed in Additional Table S2. The GAL4-ESRRBsf-LBD plasmid was mutated using Site-Directed Mutagenesis Kit QuikChange Lightning (Agilent, Santa Clara, CA, USA) and results were verified by sequencing. For the transfection assay, COS-1 cells were transfected with 2.5 μg pSG5-ERRbeta2 or pCMV-GAL4 using Trans-IT LT-1 (MirusBio, Madison, WI, USA) for 24 h. For the CAT reporter assay, COS-1 cells were transfected with 0.4 μg wild type GAL4-ESRRBsf-LBD or GAL4-ESRRBsf-LBD mutant, 4 μg of GAL-4-CAT, and 0.5 μg β-galactosidase using Trans-IT LT-1 for 24 h.

### 4.9. RNA-Seq Analysis

Total RNA was isolated from cell pellets using the TRIZOL reagent (Life Technologies, Carlsbad, CA, USA). The S4 flowcell and Paired-end 150 bp sequencing was performed on Novaseq 6000 (Illumina, San Diego, CA, USA) after an RNA-seq library was generated at Novogene Corporation Inc. (Novogene, Sacramento, CA, USA). Clean reads were aligned to human genome GRCh38.p14, and the expression level was normalized as FPKM with gene annotation file. The read count data were normalized by DESeq2 (version 1.20.0) followed by differential expression analysis. Differential expression genes and functional enrichment for Gene Ontology (GO) and the Kyoto Encyclopedia of Genes and Genomes (KEGG) were analyzed at https://us-magic.novogene.com (accessed on 2 November 2023), an online platform for data analysis and visualization provided by Novogene Corporation Inc. (Novogene, Sacramento, CA, USA), which was implemented by the edgeR R package (version 3.22.5) and the clusterProfiler R package (version 3.8.1). The raw data were uploaded to the Gene Expression Omnibus (GEO) datasets (GSE311012 1).

### 4.10. Molecular Docking

The apo (PDB: 6LN4) and holo (PDB: 6LIT) structure of the LBD of ESRRB were retrieved from the Protein Data Bank [58]. The A chains of both models were then submitted to Autodock Vina (version1.1.2) [98,99,100] to acquire a corresponding calcium or cadmium docking site. The grid box was set as Max which covers the ligand-binding pocket of ESRRB. Docking was run with or without H_2_O molecules, respectively. The structure visualization was made by Pymol (version 2.5.5).

### 4.11. Statistical Analysis

All statistical analyses were performed in Prism 10.2. Data are presented as the mean ± standard error of the mean (SEM). Statistical differences were evaluated by one-way or two-way ANOVA followed by Fisher’s LSD test or T test. Statistical significance is defined as a *p* value of <0.05: * *p* < 0.05; ** *p* < 0.01; *** *p* < 0.001; **** *p* < 0.0001.

- STAR Methods

**Table 2:** Key resource table.

Reagent/Resource	Reference or Source	Identifier or Catalog Number
**Experimental Models**		
HEK-293T cells (*H. sapiens*)	ATCC	CVCL_0063
MDA-MB-453 cells (*H. sapiens*)	Tissue Culture and Biobanking Shared Resource (Georgetown University)	CVCL_0418
COS-1 cells (*C. aethiops*)	Tissue Culture and Biobanking Shared Resource (Georgetown University)	CVCL_0223
Subcloning Efficiency DH5α Competent Cells	Invitrogen	18265-017
**Recombinant DNA**		
pSG5-ERRbeta2	Heckler & Riggins, (2015) [53]	Addgene plasmid #52186
pCMV-GAL4	Potter et al., (2010) [95]	Addgene plasmid #24345
pEQ176	Schleiss et al., (1991) [96]	Addgene plasmid #83943
pG6(5′Pro)	Taube et al., (2002) [97]	Addgene plasmid #14659
**Antibodies**		
Human ERR beta/NR3B2 Antibody	R&D systems, Minneapolis, MN, USA	PP-H6705-00
POLR2A Monoclonal Antibody (8WG16)	Thermo Fisher Scientific, Waltham, MA, USA	MA1-26249
mouse IgG control	Abcam, Cambridge, UK	ab18413 RRID: AB_2631983
goat anti-mouse IgG polyclonal antibody (594; Alexa Fluor)	Invitrogen, Waltham, MA, USA	RRID: AB_2313921
**Oligonucleotides and other sequence-based reagents**		
PCR Primers	This study	Supplemental Table S2
EsRRB Taqman probe	Thermo Fisher Scientific, Waltham, MA, USA	Hs01584024_m1
SOX2 Taqman probe	Thermo Fisher Scientific, Waltham, MA, USA	Hs01053049_s1
KLF4 Taqman probe	Thermo Fisher Scientific, Waltham, MA, USA	Hs00358836_m1
NANOG Taqman probe	Thermo Fisher Scientific, Waltham, MA, USA	Hs02387400_g1
POU5F1 (OCT4) Taqman probe	Thermo Fisher Scientific, Waltham, MA, USA	Hs00999632_g1
PRL Taqman probe	Thermo Fisher Scientific, Waltham, MA, USA	Hs00168730_m1
SFRP2 Taqman probe	Thermo Fisher Scientific, Waltham, MA, USA	Hs00293258_m1
DPT Taqman probe	Thermo Fisher Scientific, Waltham, MA, USA	Hs00355056_m1
IGF1 Taqman probe	Thermo Fisher Scientific, Waltham, MA, USA	Hs01547656_m1
ESR1 Taqman probe	Thermo Fisher Scientific, Waltham, MA, USA	Hs00174860_m1
RPLP0 Taqman probe	Thermo Fisher Scientific, Waltham, MA, USA	Hs00420895_gH
**Chemicals, Enzymes and other reagents**		
calcium chloride	Sigma-Aldrich, St. Louis, MO, USA	C4901
cadmium chloride	Sigma-Aldrich, St. Louis, MO, USA	202908
Diethylstilbestrol (DES)	Sigma-Aldrich, St. Louis, MO, USA	D4628
GSK4716	Tocris Bioscience, Briston, UK	3075
Corning^TM^ Improved Minimal Essential Medium (IMEM), phenol red-free	Corning, Corning, NY, USA	MT10026CV
fetal bovine serum (FBS)	Sigma-Aldrich, St. Louis, MO, USA	F0926
Dulbecco’s Modified Eagle’s Medium (DMEM)	Corning, Corning, NY, USA	MT10013CV
lipoic-acid and phenol-red free IMEM	Crystalgen Commack, NY, USA	226-072-12
charcoal stripped calf serum (CCS)	Sigma-Aldrich, St. Louis, MO, USA	F6765
Trizol	Life Technologies, Carlsbad, CA, USA	15596026
chloroform	Fisher Scientific, Waltham, MA, USA	J67241.K4
isopropanol	Fisher Scientific, Waltham, MA, USA	T036181000
Hank’s balanced salt solution (HBSS)	Gibco, Billings, MT, USA	14175095
Fluo-4 AM	Invitrogen, Waltham, MA, USA	F14217
formaldehyde	Fisher Scientific, Waltham, MA, USA	28908
Triton X-100	Sigma Aldrich, St. Louis, MO, USA	93443
Fisher BioReagents bovine serum albumin (BSA)	Fisher BioReagents, Waltham, MA, USA	BP9700100
Phalloidin-iFluor Conjugate (488)	AAT Bioquest, Pleasanton, CA, USA	23115
4′,6′-diamidine-2-phenylindole dihydrochloride (DAPI)	Sigma Aldrich, St. Louis, MO, USA	10236276001
PhosSTOP/PI cocktail	Sigma Aldrich, St. Louis, MO, USA	4906837001 and 4693159001
Nuclei lysis buffer	Sigma Aldrich, St. Louis, MO, USA	20-163
Chromatin Immunoprecipitation Dilution Buffer	Sigma Aldrich, St. Louis, MO, USA	20-153
Low Salt Immune Complex Wash Buffer	Sigma Aldrich, St. Louis, MO, USA	20-154
High Salt Immune Complex Wash Buffer	Sigma Aldrich, St. Louis, MO, USA	20-155
LiCl Immune Complex Wash Buffer	Sigma Aldrich, St. Louis, MO, USA	20-156
PCR purification kit (QIAquick PCR purification kit)	QIAGEN, Germantown, MD, USA	28106
SYBR green mastermix (PowerUP SYBR Green Master Mix)	Thermo Fishier Scientific, Waltham, MA, USA	A25780
BspDI Restriction Enzyme	New England Biolabs, Ipswich, MA, USA	R0557
NotI-HF Restriction Enzyme	New England Biolabs, Ipswich, MA, USA	R3189
Taq 2X Master Mix	New England Biolabs, Ipswich, MA, USA	M0270L
T4 DNA Ligase	New England Biolabs, Ipswich, MA, USA	M0202
Maxima H MA Synthesis Master Mix with dsDNase	Thermo scientific, Waltham, MA, USA	M1681
PureLink^TM^ HiPure Plasmid Maxiprep Kit	Invitrogen, Waltham, MA, USA	K210007
QuikChange Lightning Site-Directed Mutagenesis Kit	Agilent, Santa Clara, CA, USA	210518
Trans-IT LT-1	MirusBio, Madison, WI, USA	MIR 2306
SsoAdvanced Universal Probes Supermix	Bio-Rad, Hercules, CA, USA	172-5281
**Software**		
GraphPad Prism version 10.2	https://www.graphpad.com (accessed on 27 March 2024).	
ImageJ version 1.53p	https://imagej.nih.gov/ij/index.html (accessed on 16 June 2021)	
UCSC Genome Browser	https://genome.ucsc.edu/ (accessed on 20 August 2017)	
Cistrome Data Browser	http://cistrome.org/db/#/ (accessed on 11 September 2017)	
QuikChange Primer Design of Agilent	https://www.agilent.com/store/primerDesignProgram.jsp (accessed on 10 December 2017)	
Novo Magic	https://us-magic.novogene.com (accessed on 31 October 2023)	
Autodock Vina version 1.1.2	https://vina.scripps.edu/ (accessed on 13 July 2023) [98,99,100]	
Pymol version 2.5.5	https://www.pymol.org/ (accessed on 11 April 2023)	
**Other**		
fluorimeter	Photon Technology International, Lawrenceville, NJ, USA	
Olympus IX71 microscope	Olympus, Tokyo, Japan	
SP8 laser confocal microscope	Leica, Wetzlar, Germany	
Novaseq 6000	Illumina, San Diego, CA, USA	

## Figures and Tables

**Table 1:** Docking result of Ca/Cd interaction sites on the LBD of ESRRB.

No.	Protein	Metal	H_2_O	Affinity (Kcal/mol)	RMSD	Amino Acid in Interaction	Chains	Helix
1	6LN4 (Apo)	Ca/Cd	Yes	−1.5	0	S278, E282		H4, H5
2	6LN4 (Apo)	Ca/Cd	Yes	−1.3	14.67	G270, D271, S274, Y356		H4
3	6LN4 (Apo)	Ca/Cd	No	−1.3	9.499	G270, D271, S274, Y356		H4
4	6LN4 (Apo)	Ca/Cd	No	−1.2	7.908	S274, S278		H4, H4–5
5	6LN4 (Apo)	Cd	Yes	−1.1	3.956	Q277, S278		H4, H4–5
6	6LIT (Holo)	Ca	Yes	−1.5	30.673	S278	dimer-A	H4–5
7	6LIT (Holo)	Ca/Cd	Yes	−1.5	0–7.292	S278	monomer	H4–5
8	6LIT (Holo)	Ca/Cd	Yes	−1.5	29.55–30.966	Q277, S278	dimer-A/B	H4, H4–5
9	6LIT (Holo)	Ca/Cd	Yes	−1.5	1.349–10.131	Q277, S278	monomer	H4, H4–5
10	6LIT (Holo)	Ca/Cd	Yes	−1.4	24.96–27.243	S278, S352	dimer-A	H4–5, H8–9
11	6LIT (Holo)	Ca/Cd	Yes	−1.4	6.203–7.296	S278, S352	monomer	H4–5, H8–9
12	6LIT (Holo)	Ca	Yes	−1.5	27.288	Q401	dimer-B	H10 + H11
13	6LIT (Holo)	Cd	No	−1.3	22.767	E427, L429, E430	monomer	H12

RMSD: root mean square difference between the docker complex and the input structure.

## Data Availability

The original contributions presented in this study are included in the article/Supplementary Material. Further inquiries can be directed to the corresponding author. The RNA-seq data discussed in this publication have been deposited in NCBI’s Gene Expression Omnibus and are accessible through GEO Series accession number GSE.

## Supplementary Materials

The following supporting information can be downloaded at: https://www.mdpi.com/article/10.3390/ijms27010231/s1.

## Abbreviations

AF-1 or AF-2	activation function-1 or -2
CAT	chloramphenicol acetyltransferase
CCS	charcoal-stripped calf serum
ChIP	Chromatin Immunoprecipitation Assay
CSCs	cancer stem cells
DAPI	4, 6-diamidino-2-phenylindole
DBD	DNA-binding domain
DEGs	differentially expressed genes
DES	diethylstilbestrol
DMEM	Dulbecco’s Modified Eagle’s Medium
DPT	dermatopontin, tyrosin-rich acidic matrix protein
ERRE	estrogen-related receptor response elements
ESR	estrogen receptor
ESR1 or ERα	estrogen receptor alpha
ESRRA	estrogen-related receptor alpha
ESRRB-∆10	ESRRB splice variant delta 10
ESRRB-sf	ESRRB splice variant short form
ESRRB	estrogen-related receptor beta
ESRRB2	ESRRB splice variant beta 2
ESRRG	estrogen-related receptor gama
ESRRs	estrogen-related receptors
FBS	fetal bovine serum
FoxO	forehead box protein, member of class O
GAL4	galectin 4
GO	Gene Ontology
HBSS	Hank’s balanced salt solution
HER2	human epidermal growth factor 2
IF	immunofluorescence
IGF1	insulin-like growth factor 1
IMEM	improved minimal essential medium
KEGG	Kyoto Encyclopedia of Genes and Genomes
KLF4	Kruppel-like factor 4
LBD	ligand-binding domain
MAPK	mitogen-activated protein kinase
MaSCs	mammary stem cells
NANOG	Nanog homeobox
OCT4	OCT3/4 or POU class 5 homeobox1
PGR	progesterone receptor
Pol2	RNA polymerase 2
PRL	prolactin
Re-ChIP	subsequential Chromatin Immunoprecipitation Assay
real time-qPCR	real-time polymerase chain reaction
RMSF	Root mean square fluctuation
RPLP0	ribosomal protein P0
RXRα	Retinoid X receptor-alpha
SFRP2	secreted frizzle protein 2
SOX2	sex-determining region Y-box 2
TFs	transcription factors
TGFβ	tumor growth factor-beta
TNBC	triple negative breast cancer
TSS	transcription start site
