# Supplementary material for: Calcium and Cadmium Activate ESRRB to Mediate Cell Stemness and Pluripotency
Source: Int J Mol Sci. 2025 Dec 25;27(1):231. doi: 10.3390/ijms27010231 (PMC12785951; doi:10.3390/ijms27010231)
Supplement: Supplementary file 1 [file ijms-27-00231-s001.zip › ijms-3856060-supplementary.pdf]

Supplementary Materials:

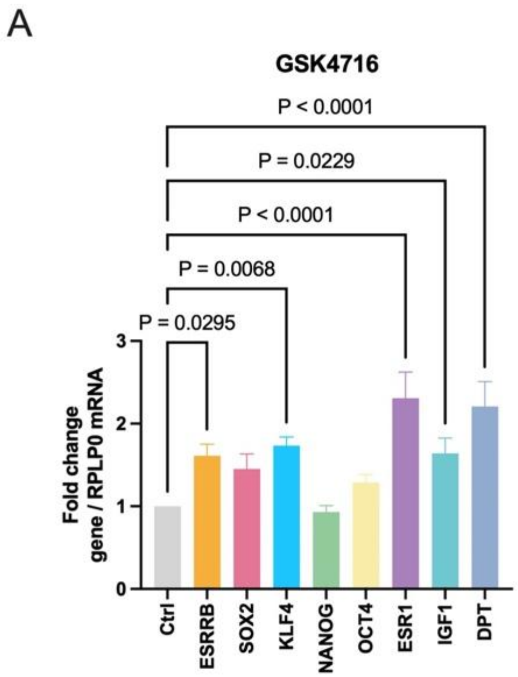

Supplemental Figure S1. Effects of GSK4716 on ESRRB regulated genes in MDA-MB-453 cells.

MDA-MB-453 cells were treated with GSK4716 (GSK4716; 5 μM) for 24 hours in serum-free medium.

A, Effects of GSK4716 on the ESRRB target genes.

Data are expressed as fold change (mean ± SEM); n= 3 to 5; statistical significance is defined as a P value of ≤ 0.05, P value is marked above if statistically significant.

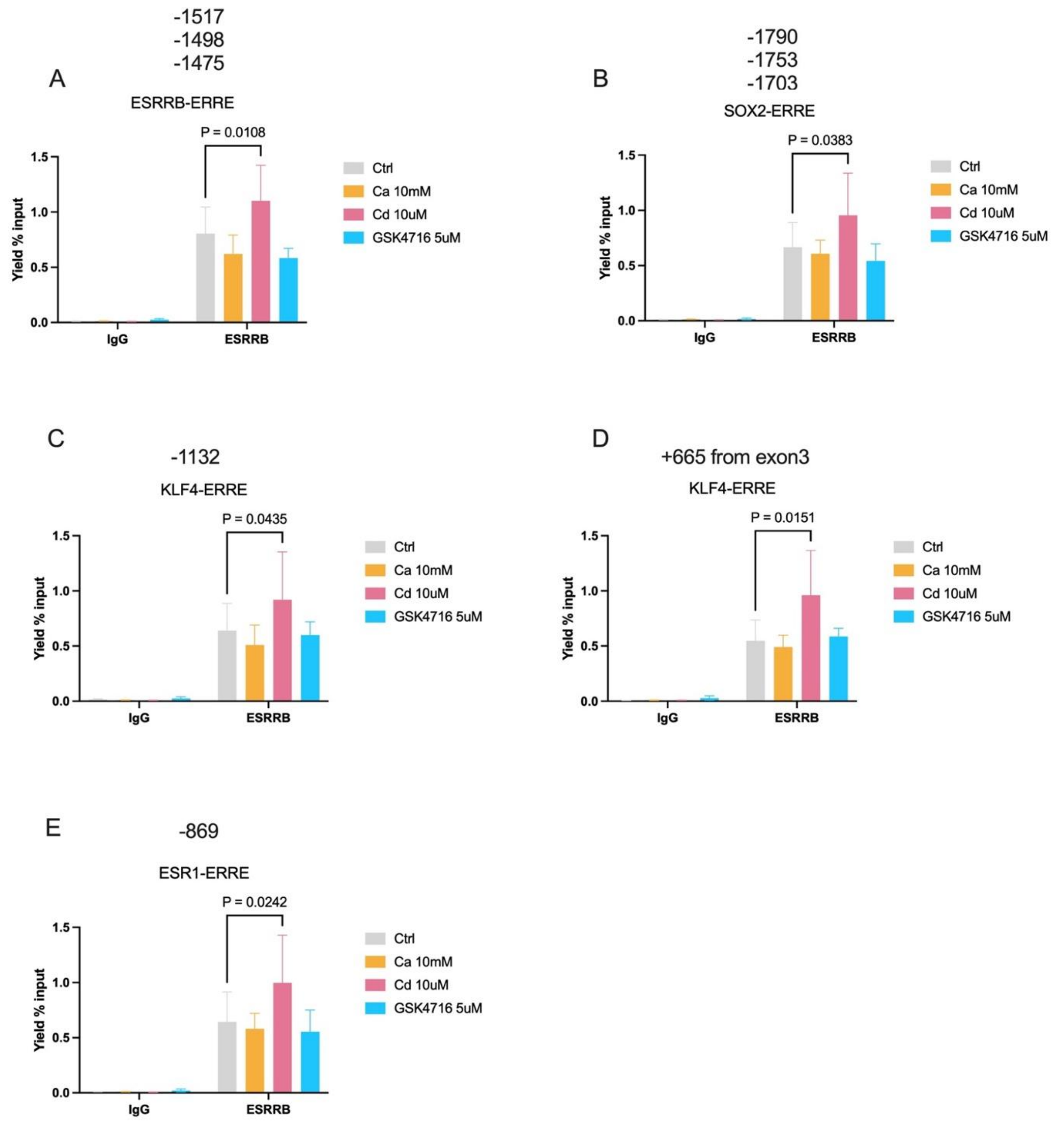

Supplemental Figure S2. Effects of calcium and cadmium on the recruitment of ESRRB to ERREs of ESRRB regulated genes in MDA-MB-453 cells.

A-H, effect of calcium, cadmium, and GSK4716 on recruitment of ESRRB to the ERREs of *ESRRB* (A), *SOX2* (B), *KLF4* upstream enhancer (C) and intron enhancer (D), and *ESR1* (E). The numbers above each figure indicate the location of ERREs of target genes. Data are expressed as fold change compared to control in % input (mean ± SEM); n=2 to 3; statistical significance is defined as a P value of ≤ 0.05, P value is marked above if statistically significant.

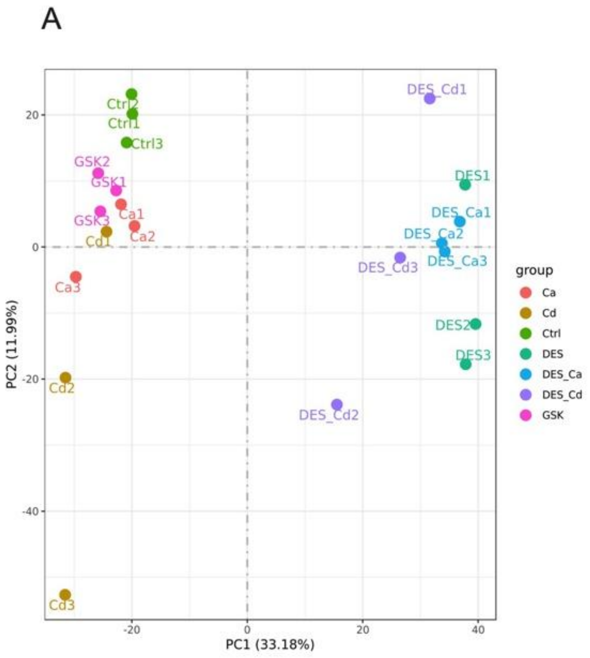

Supplemental Figure S3. Effects of calcium and cadmium on cell pluripotency.

A, principal component analysis (PCA) clustering graph of samples in groups.

Supplemental Table S1. List of DEGs only in Ca Vs Ctrl, Cd Vs Ctrl and GSK Vs Ctrl

Up regulated: 40			*Down regulated: 37*		
No.	ENSEMBL ID	Gene	No.	ENSEMBL ID	Gene
1	ENSG00000128917	DLL4	1	ENSG00000143476	DTL
2	ENSG00000148926	ADM	2	ENSG00000094804	CDC6
3	ENSG00000136999	NOV	3	ENSG00000168496	FEN1
4	ENSG00000129116	PALLD	4	ENSG00000062822	POLD1
5	ENSG00000108551	RASD1	5	ENSG00000283526	PRRT1B
6	ENSG00000153714	LURAP1L	6	ENSG00000132646	PCNA
7	ENSG00000087074	PPP1R15A	7	ENSG00000165244	ZNF367
8	ENSG00000078018	MAP2	8	ENSG00000198554	WDHD1
9	ENSG00000119686	FLVCR2	9	ENSG00000182575	NXPH3
10	ENSG00000196935	SRGAP1	10	ENSG00000166508	MCM7

11	ENSG00000143507	DUSP10	11	ENSG00000076003	MCM6
12	ENSG00000092969	TGFB2	12	ENSG00000117748	RPA2
13	ENSG00000196352	CD55	13	ENSG00000176890	TYMS
14	ENSG00000162772	ATF3	14	ENSG00000125885	MCM8
15	ENSG00000184545	DUSP8	15	ENSG00000112029	FBXO5
16	ENSG00000020577	SAMD4A	16	ENSG00000118655	DCLRE1B
17	ENSG00000163513	TGFBR2	17	ENSG00000188985	DHFRP1
18	ENSG00000166928	MS4A14	18	ENSG00000159259	CHAF1B
19	ENSG00000146592	CREB5	19	ENSG00000175785	PRIMA1
20	ENSG00000233930	KRTAP5-AS1	20	ENSG00000078900	TP73
21	ENSG00000147883	CDKN2B	21	ENSG00000080839	RBL1
22	ENSG00000153721	CNKSR3	22	ENSG00000049541	RFC2
23	ENSG00000171867	PRNP	23	ENSG00000198056	PRIM1
24	ENSG00000070540	WIPI1	24	ENSG00000174871	CNIH2
25	ENSG00000205189	ZBTB10	25	ENSG00000123219	CENPK
26	ENSG00000006652	IFRD1	26	ENSG00000175305	CCNE2
27	ENSG00000065809	FAM107B	27	ENSG00000181938	GINS3
28	ENSG00000154734	ADAMTS1	28	ENSG00000014138	POLA2
29	ENSG00000140941	MAP1LC3B	29	ENSG00000051341	POLQ
30	ENSG00000158406	HIST1H4H	30	ENSG00000163072	NOSTRIN
31	ENSG00000106070	GRB10	31	ENSG00000123416	TUBA1B
32	ENSG00000157873	TNFRSF14	32	ENSG00000115687	PASK
33	ENSG00000276248	AL442125.1	33	ENSG00000285043	ALDOA
34	ENSG00000136052	SLC41A2	34	ENSG00000146674	IGFBP3
35	ENSG00000167995	BEST1	35	ENSG00000163362	INAVA
36	ENSG00000171124	FUT3	36	ENSG00000134323	MYCN
37	ENSG00000147894	C9orf72	37	ENSG00000105173	CCNE1
38	ENSG00000073910	FRY			
39	ENSG00000132692	BCAN			

40	ENSG00000229953	AL590666.2		

List includes both ENSEMBL ID and their corresponding gene names in up regulated and down regulated groups.

Supplemental Table S2. Primer sequence

Experiment	Gene/ purpose	Primer sequence
ChIP/ re-ChIP	ESRRB-ERRE-2	Forward: CTG CTG TGT GCC TGG AGA AT
		Reverse: GAA AGA GGG GTG CAG GGA TG
	ESRRB-ERRE-5	Forward: CCA CCC CAG CTT GAT TCT GT
		Reverse: GGA GCC CAC TAA CCC TAC TG
	SOX2-ERRE-3	Forward: AGT CTG ATC TTC CAT CCC CCT CT
		Reverse: CCC CCT GCT GGT AGA TTC GC
	SOX2-ERRE-4	Forward: GGG GGA GTG ATT ATG GGA AGA AGG
		Reverse: CTC CTC CCC TGG TCT ACC CTT AC
	KLF4-ERRE-up-2	Forward: TTA CAG GCA GTG GCT CAC G
		Reverse: TTT TCG TGG AGA CGG GGT TT
	KLF4-ERRE-in-2	Forward: AGT CGT TTA GGT GCA GCA TT
		Reverse: TTG TGA TTG GTA GTG TGC CCA A
	ESR1-ERRE-2	Forward: ACC GAC AAT GTA ACA TAA TTG CCA
		Reverse: ATG CCT TCC ACA GGT TGG TTA
	ESR1-ERRE-5	Forward: AGC TGC TCT TTG GGA TCG CT
		Reverse: TAC AAA GGT GCT GGA GGA CGG
Gal4-ESRRBsf-LBD	ESRRB2-LBD cloning	Forward: GCG TCA TGA TCT GAA GGA AGG TGT GCG CCT TG
		Reverse: GAA GCG GCC GCT CAC ACC TTG GCC TCC
	LDB delete 434-500	Forward: AAG ATC TGG ATC CTC AGG CCT TGG CTT CCA GC
		Reverse: GCT GGA AGC CAA GGC CTG AGG ATC CAG ATC ATC TT
	A433V mutation	Forward: GAT CTG GAT CCT CAG ACC TTG GCT TCC AGC A
		Reverse: TGC TGG AAG CCA AGG TCT GAG GAT CCA GAT C
	ESRRBsf-LBD-BspD1	Forward: TGA CTG TAT CGA TTC TGA AGG AAG GTG TGC GCC TTG
	ESRRBsf-LBD-Not1	Reverse: GAA GCG GCC GCT CAC ACC TTG GCC TCC AGC ATC T
CAT reporter	CAT	Forward: TTC TTG CCC GCC TGA TGA AT
		Reverse: ACC GTA ACA CGC CAC ATC TT
	β-galactosidase	Forward: ATG GGT AAC AGT CTT GGC GG
		Reverse: GGC GTA TCG CCA AAA TCA CC
Mutagenesis	S274A	Forward: GGG CGA CCA GAT GGC CCT GCT CCA GAG C
		Reverse: GCT CTG GAG CAG GGC CAT CTG GTC GCC C

	S278A	Forward: GAT GTC CCT GCT CCA GGC CGC TTG GAT GGA GAT C
		Reverse: GAT CTC CAT CCA AGC GGC CTG GAG CAG GGA CAT C
	Q401A	Forward: CCC TGC TGA GGG CAA CCG CTG CCA AGG
		Reverse: CCT TGG CAG CGG TTG CCC TCA GCA GGG
	Q408A	Forward: CTG CCA AGG CCG TGG CGC ACT TCT ACA GCG
		Reverse: CGC TGT AGA AGT GCG CCA CGG CCT TGG CAG
	Q416A	Forward: CTA CAG CGT GAA GCT GGC GGG AAA AGT GCC CAT GC
		Reverse: GCA TGG GCA CTT TTC CCG CCA GCT TCA CGC TGT AG
	E427A	Forward: GCA CAA GCT GTT CCT GGC GAT GCT GGA AGC CAA GG
		Reverse: CCT TGG CTT CCA GCA TCG CCA GGA ACA GCT TGT GC
	E430A	Forward: CTC ACA CCT TGG CTG CCA GCA TCT CCA GG
		Reverse: CCT GGA GAT GCT GGC AGC CAA GGT GTG AG
